# RanBP9 Plays a Critical Role in Neonatal Brain Development in Mice

**DOI:** 10.1371/journal.pone.0066908

**Published:** 2013-06-26

**Authors:** Juan Pablo Palavicini, Brandon Noel Lloyd, Crystal D. Hayes, Elisabetta Bianchi, David E. Kang, Ken Dawson-Scully, Madepalli K. Lakshmana

**Affiliations:** 1 Section of Neurobiology, Torrey Pines Institute for Molecular Studies, Port Saint Lucie, Florida, United States of America; 2 Department of Biological Sciences, Charles E. Schmidt College of Science, Florida Atlantic University, Boca Raton, Florida, United States of America; 3 Laboratory of Immuneregulation, Department of Immunology, Institut Pasteur, Paris, France; 4 Department of Molecular Medicine, USF Health Byrd Alzheimer’s Institute, Tampa, Florida, United States of America; Tokyo Medical and Dental University, Japan

## Abstract

RanBP9 is known to act as a scaffolding protein bringing together a variety of cell surface receptors and intracellular targets thereby regulating functions as diverse as neurite and axonal outgrowth, cell morphology, cell proliferation, myelination, gonad development, myofibrillogenesis and migration of neuronal precursors. Though RanBP9 is ubiquitously expressed in all tissues, brain is one of the organs with the highest expression levels of RanBP9. In the neurons, RanBP9 is localized mostly in the cytoplasm but also in the neurites and dendritic processes. We recently demonstrated that RanBP9 plays pathogenic role in Alzheimer’s disease. To understand the role of RanBP9 in the brain, here we generated RanBP9 null mice by gene-trap based strategy. Most of Ran−/− mice die neonatally due to defects in the brain growth and development. The major defects include smaller cortical plate (CP), robustly enlarged lateral ventricles (LV) and reduced volume of hippocampus (HI). The lethal phenotype is due to a suckling defect as evidenced by lack of milk in the stomachs even several hours after parturition. The complex somatosensory system which is required for a behavior such as suckling appears to be compromised in Ran−/− mice due to under developed CP. Most importantly, RanBP9 phenotype is similar to ERK1/2 double knockout and the neural cell adhesion receptor, L1CAM knockout mice. Both ERK1 and L1CAM interact with RanBP9. Thus, RanBP9 appears to control brain growth and development through signaling mechanisms involving ERK1 and L1CAM receptor.

## Introduction

Ran-binding protein 9 (RanBP9) also called RanBPM is a multi-modular scaffolding protein implicated in a variety of functions through integration of cell surface receptors with intracellular signaling targets [1], [2]. RanBP9 is well conserved in organisms at all levels of evolution starting from xenopus to human. Sequence homology search using prosite identifies several conserved domains implicated in a variety of functions. At the most N-terminal region, there is a proline-rich region [3], which is important for both very rapid and remarkably strong but less specific protein binding. Next is the SPRY domain (spore lysis A and the ryanodine receptor), which is further differentiated into less conserved region, PRY followed by the highly conserved SPRY domain [4]. The LisH (Lissencephaly type-1 like homology) domain has been implicated in protein dimerization or oligomerization [5], [6]. The CTLH (C-terminal to LisH) domain function is unknown. Finally, the CRA (CT11-RanBP9) domain at the c-terminal end of RanBP9 is also a protein-protein interaction domain shown to bind fragile X mental retardation protein (FMRP) in the microtubule organizing center [7].

Although RanBP9 is ubiquitously expressed in all tissues, its protein levels are relatively higher in the brain, heart and skeletal muscles [8], [9]. The subcellular localization of RanBP9 varies depending on the cell type and differentiation state. For example, in rapidly dividing cells, RanBP9 appears to be mostly localized to the cytosol and nucleus, whereas in more differentiated cells (*i.e.*, Madin-Darby canine kidney cells or muscle), the vast majority of RanBP9 is found discretely near the inner surface of the plasma membrane or associated with cytoskeletel elements [10]–[13]. RanBP9 is also known to exist as a component of large protein complex which could be both cytoplasmic and nuclear [14], [15]. This type of differential subcellular localization is consistent with the postulated role of RanBP9 as a multifunctional scaffolding protein that interacts with cytoplasmic domains of a variety of membrane receptors such as integrin β subunit [11], c-Met [9], L1CAM [16], CD39 [17] and the calcium channel, Ca_v_3.1 [18], mediating diverse transmembrane signaling.

Several protein interactions with RanBP9 also suggest a critical role for RanBP9 in the brain. RanBP9 interaction with plexin-A mediates or regulates semaphorin3A signaling controlling axonal outgrowth [19]. In addition, RanBP9 not only interacts with protein-tyrosine kinase receptor MET, Axl/Sky, TrkA, but also with the receptor tyrosine kinase tropomyosin-related kinase B (TrkB) which serves as the receptor for brain-derived neurotrophic factor (BDNF) and regulates BDNF-mediated neuronal morphology and survival through the MAPK and Akt pathways [20]. Indeed, using RNAi in utero, Chang et al [21], demonstrated that RanBP9 regulates the progression of neuronal precursors through M-phase and decreased the number of cells in cytokinesis. This study clearly suggested an important role for RanBP9 in the brain development. Taken together, diverse roles of RanBP9 in several signal transduction pathways clearly indicates a compelling role for RanBP9 in brain development.

Here we generated RanBP9 knockout mice (Ran−/−) and found that RanBP9 is crucial for neonatal brain development and survival. Ran−/− pups displayed reduced volumes of major brain regions such as cortex and hippocampus. Consequently, the volumes of lateral ventricles were robustly enlarged due to striking reduction in cell density.

## Results

### Characterization of RanBP9 Null Mice

**Figure 1:**
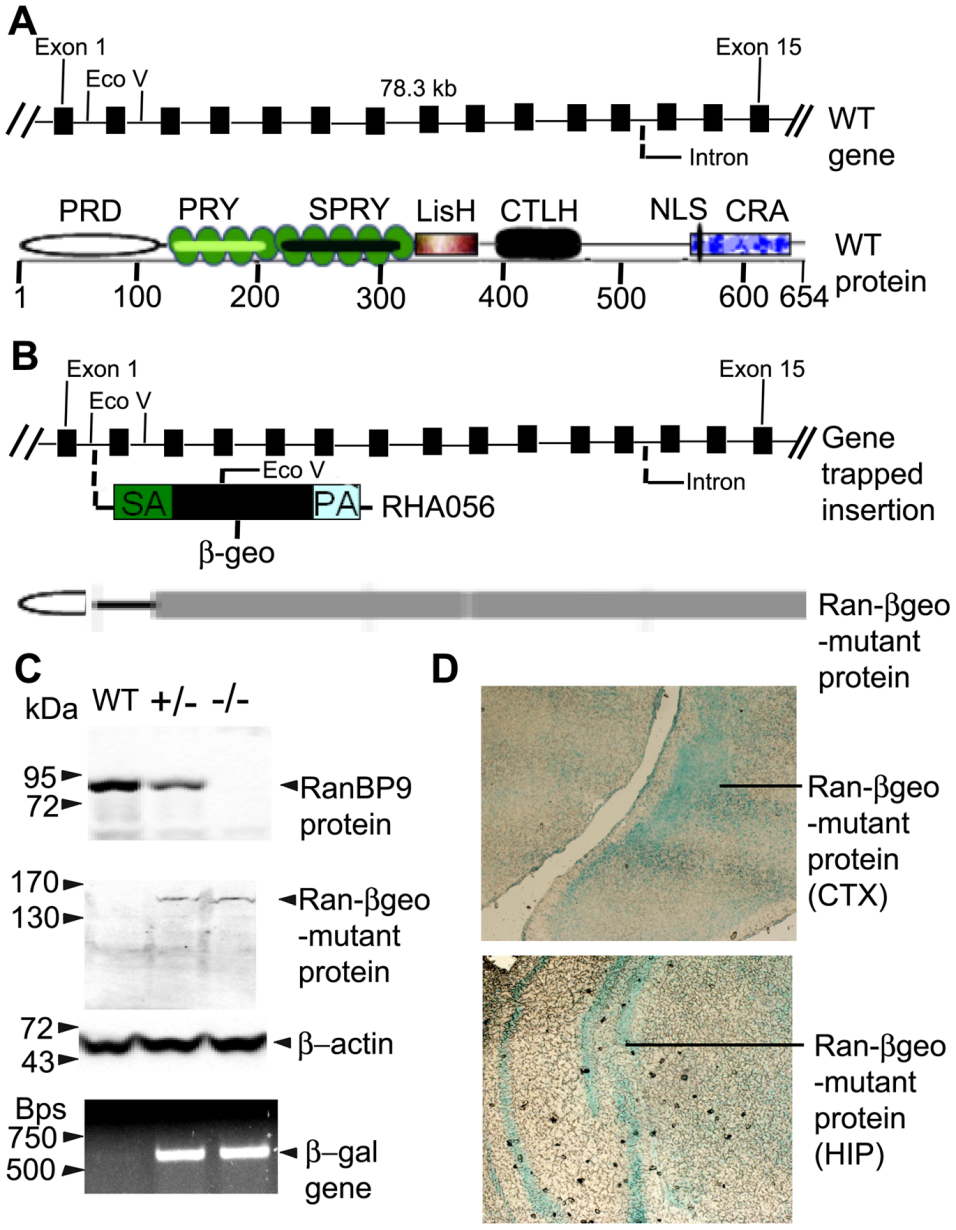
Targeted disruption of RanBP9 gene by gene trap strategy. (A), Schematic view of the exon structure and the restriction map of RanBP9 wild-type (WT) gene (top) and the domain organization of the predicted WT protein (bottom). (B) Schematic view of the βgeo cassette trapped between exon 1 and 2 of RanBP9 gene (top) and the Ran-βgeo mutant protein (bottom). (C), Western blot analysis of P1 brain lysates showing RanBP9 protein levels completely absent in Ran−/− and about 50% levels in Ran+/− mice compared to WT mice (top panel). Western blots detection of Ran-βgeo mutant protein by β-gal protein specific antibody (middle panel). PCR amplifications of βgeo gene by primers targeted against β-gal gene (bottom), confirming successful integration of the βgeo cassette within RanBP9 gene. (D), Immunohistochemical detection of the expression of Ran-βgeo mutant protein in the cortex (top) and the hippocampus (bottom).

The germ line transmission of the mutated RanBP9 allele was initially confirmed by PCR amplification of β-gal gene from the total genomic DNA extracted from the tails using the forward primer, 5′-TTA TCG ATG AGC GTG GTG GTT ATG C-3′ and the reverse primer, 5′-GCG CGT ACA TCG GGC AAA TAA TAT C-3′. The PCR reaction reliably amplified a 700 bp fragment within the β-galactosidase gene (Fig. 1C, bottom panel). The β-galactosidase enzyme activity was also confirmed on coronal brain sections by staining with X-gal. A moderate β-gal staining in the heterozygous mouse brains and robust staining in the homozygous mouse brains was observed (Fig. 1D, shown for cortex and hippocampus). We also stained the whole body of pups with X-gal and confirmed the genotype-dependent blue color intensity which was not observed in WT pups. But because β-gal activity alone does not indicate whether the RanBP9 gene is mutated, we next wanted to confirm RanBP9 gene inactivation by immunoblots. We confirmed about 50% reduction in the levels of RanBP9 protein in the heterozygous mouse brains and complete absence in the homozygous mouse brains in many neonatal mice by immunoblots using RanBP9 specific monoclonal antibody (Fig. 1C, upper panel). Additionally, because successful inactivation of trapped gene is expected to produce Ran-βgeo mutant fusion protein, we re-probed the blots and confirmed the expression of high molecular weight Ran-βgeo mutant fusion protein detected by an antibody that specifically recognizes β-galactosidase (Fig. 1C, middle panel) suggesting that the complete absence of RanBP9 protein is the result of the mutant fusion protein produced by gene-trapping. These results confirm and establish successful production of RanBP9 null mice.

### Significant Reduction of Body and Brain Weights in Ran−/− Pups

**Figure 2:**
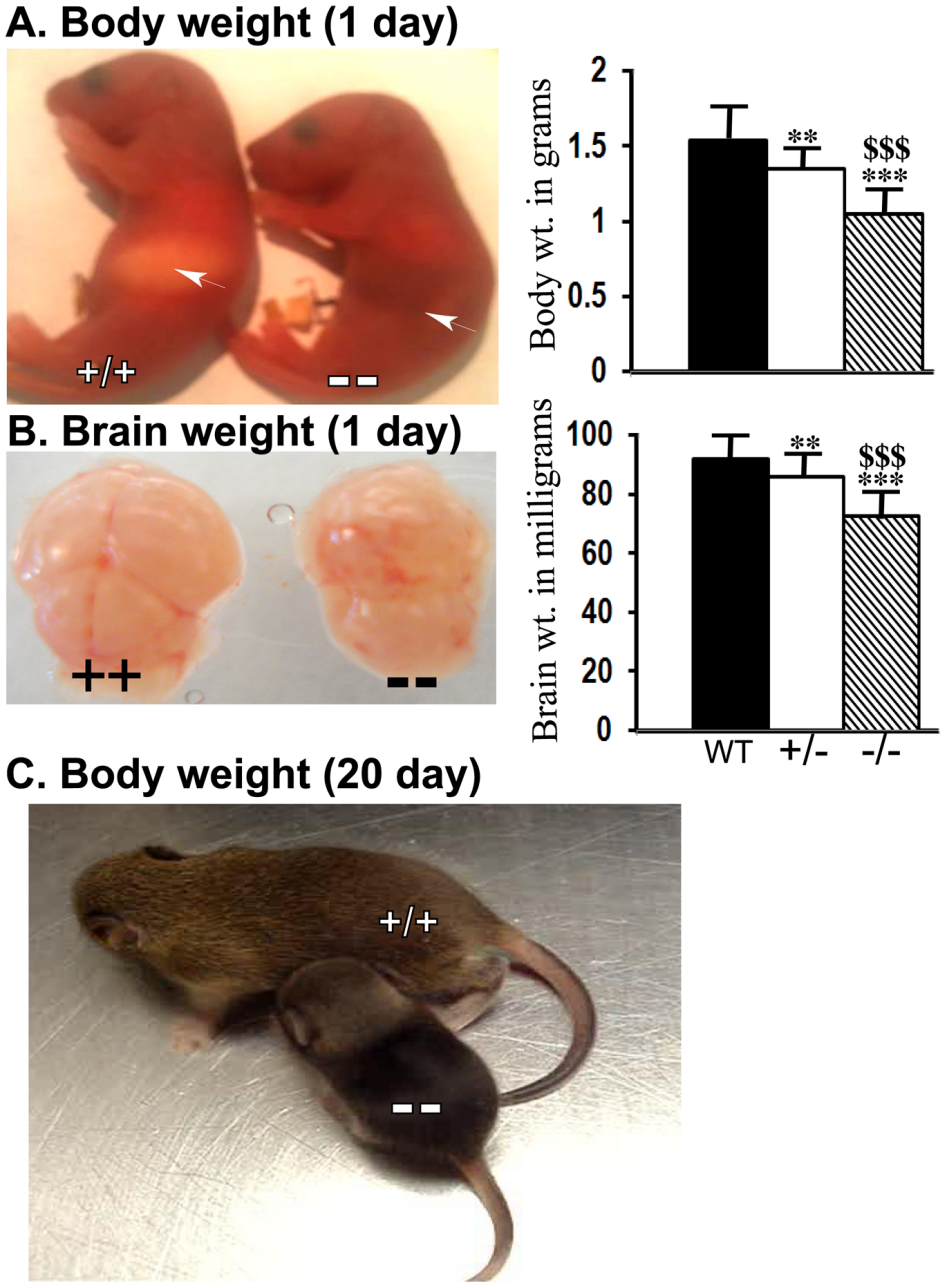
Behavioral as well as body and brain weight differences between WT, Ran+/− and Ran−/− mice. Due to defects in suckling, Ran−/− mice fail to drink milk (arrow), whereas WT mice are filled with white milk. (A), A representative WT (+/+) and littermate Ran−/− (−/−) P1 mice are shown. The histogram on the right shows reduced body weights by 12% in Ran+/− mice and by 31% in Ran−/− mice compared to WT littermates. (B), Representative brain pictures are shown for WT and littermate Ran−/− P1 mice. Histograms on the right shows reduced brain weights by 6% in Ran+/− and by 21% in Ran−/− mice compared to littermate WT mice. Body weights are expressed in grams and brain weights in milligrams. (C) A picture of typical WT and one of very few Ran−/− mice that survived until three weeks of age. One-way analysis of variance (ANOVA) followed by post-hoc Tukey-Kramer multiple comparisons test revealed significant differences. In each group n = 6, ±SEM. **, p<0.01, ***, p<0.001 when WT was compared with Ran+/− or Ran−/− mice. $$$, p<0.001 when Ran+/− mice was compared with Ran−/− mice.

Careful monitoring of Ran−/− pups in multiple litters revealed that most Ran−/− pups survived until postnatal day 1(P1, considering day of birth as day 0). In our experience very few pups survived to a maximum of three weeks. Most of the Ran−/− pups died within 24 hours. The death seems to be caused by failure to latch and suckle milk as can be judged from the complete absence of milk in the Ran−/− pups compared to littermate WT pups whose stomach is filled with white milk (Fig. 2A, indicated by an arrow). Another recent study which produced RanBP9 null mice using the same strategy as ours also reported that most Ran−/− pups died immediately after birth, though some mice survived to adult hood albeit with reduced body weight [22]. But in our hand almost all pups died by postnatal day 2. The reasons for these differences are unknown. We also noticed a 12% and 31% reduction (Fig. 2A, p<0.01) in the body weights of Ran+/− and Ran−/− pups respectively compared to WT littermates (Fig. 2A, p<0.001). Similarly we noticed significant differences in brain weights in Ran+/− mice by 6% (Fig. 2B, p<0.01) and in Ran−/−mice by 21% (Fig. 2B, p<0.001). Few mice that survived to about three weeks showed a two-fold reduction in body weight (Fig. 2C) compared to WT littermates. Interestingly, these mice showed striking differences in their gait and hind limb coordination during locomotion. Immediately after birth, the pups looked reddish and normal in every aspect suggesting that the neonatal lethality does not result from defects in either cardiovascular or respiratory systems. Inability to suckle milk is expected to lead to death for not only lack of nourishment but also for loss of bodily homeostasis in the absence of any liquid derived from the milk. Inability to suckle may indirectly indicate abnormality in brain development including somatosensory systems as well as defects in either neuromuscular or craniofacial development. Many neonatal lethal mutations have been identified in mice that are associated with an inability to suckle milk [23].

### RanBP9−/− Mice Show Severe Defects in Neonatal Brain Development

**Figure 3:**
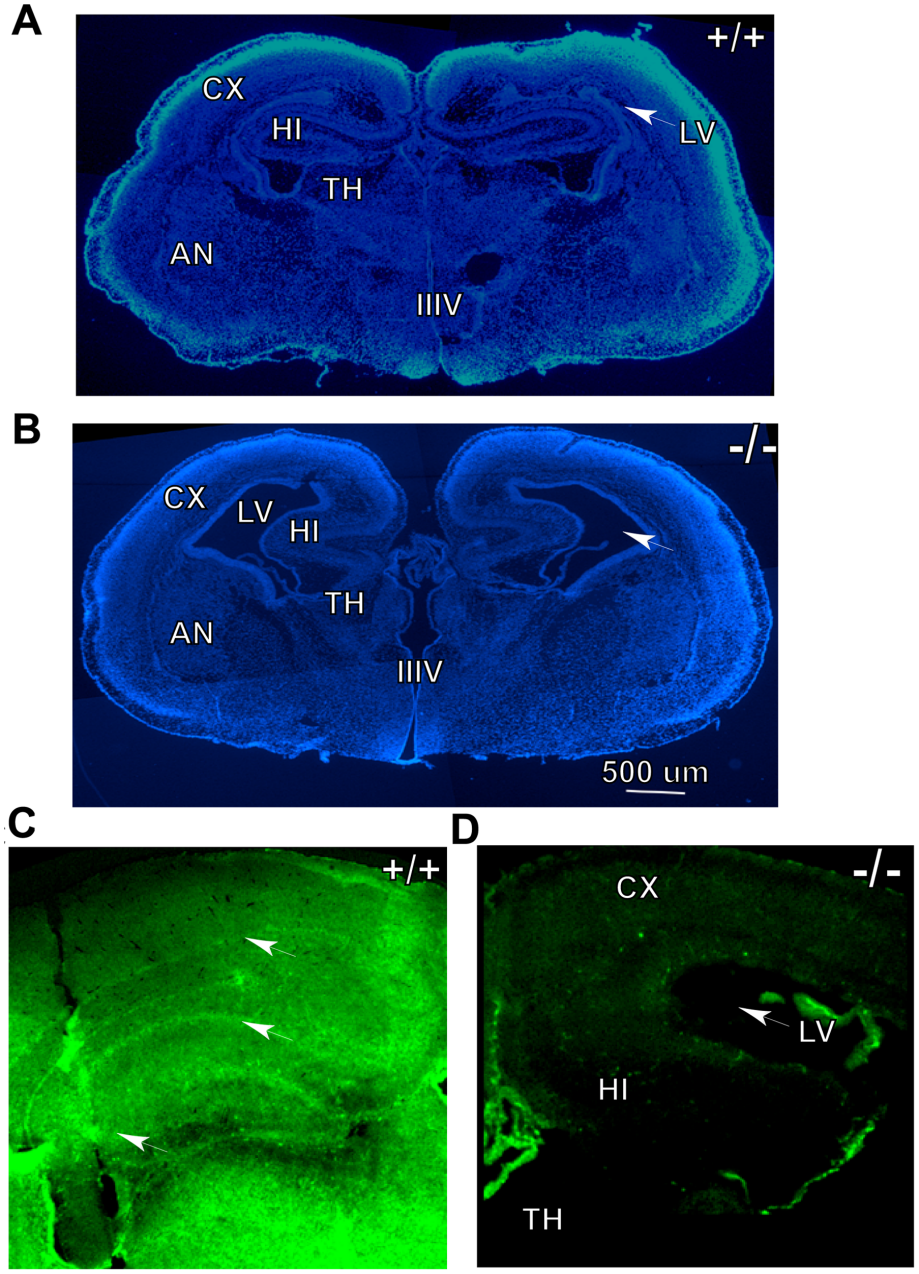
DAPI-stained brain sections from WT (A) and Ran−/− (B) P1 mice showing gross anatomical differences. Of particular note is the robustly enlarged lateral ventricular volume (arrow) in Ran−/− brain compared to WT brain which has negligible size of the lateral ventricle at this developmental stage. (C) Immunohistochemical staining of neonatal WT P0 brain with RanBP9-specific antibody shows RanBP9 immunoreactivity in all layers of the cortex as well as hippocampus, whereas in Ran−/− brains such immunoreactivity is completely absent (D). CX, cortex; LV, lateral ventricle; HI, hippocampus; TH, thalamus; IIIV, the third ventricle and AN, amygdaloid nuclei.

**Figure 4:**
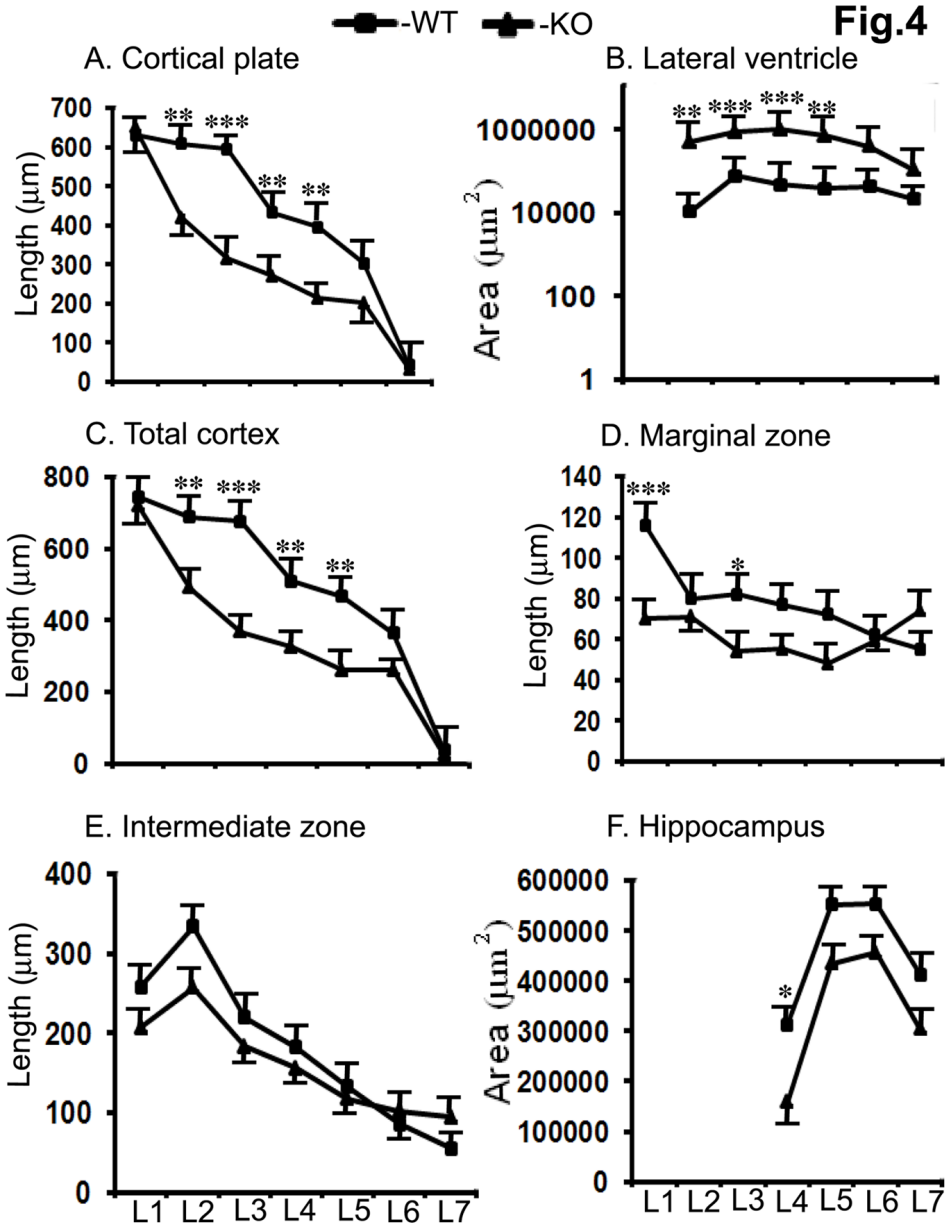
Quantitation of length and total region areas of the DAPI-stained brain sections of P1 mice showed significant differences between WT and Ran−/− mice. Brain sections from level 1 (L1) to level 7 (L7) corresponding to the sections in the Electronic Prenatal Mouse Brain Atlas (EPMBA) were analyzed. The most affected brain area was the volume of the lateral ventricle which was significantly increased in Ran−/− brains at L2 (46-fold), L3 (10-fold), L4 (19-fold) and L5 (17-fold). The cortical plate showed significant decrease in the length at L2 (31%), L3 (47%), L4 (38%) and L5 (46%). The length of total cortex (marginal zone+cortical plate+intermediate zone) was also significantly reduced in Ran−/− brains at L2 (29%), L3 (46%), L4 (36%) and L5 (44%). Marginal zone was decreased only at L1 (39%) and L3 (34%) and the hippocampus only at L4 (49%), but intermediate zone was not affected in Ran−/− brains. One-way ANOVA followed by post-hoc Bonferroni multiple comparisons test revealed significant differences. In each group n = 6, ±SEM. *, p<0.05, **, p<0.01, ***, p<0.001in Ran−/− brains compared to littermate WT controls.

We next examined Ran−/− pups in detail by histochemistry as well as immunohistochemistry to see if there are any overt defects in the anatomy of the developing brain. We initially treated P1 brain sections with DAPI to stain all cellular nuclei to visualize cell density throughout the brain. We noticed gross anomalies especially in cortical plate development which plausibly account for the neonatal lethality due to inadequate somatosensory or motor development which regulate feeding reflexes. Another striking difference that we noticed in Ran−/− pups compared to WT littermates is robustly enlarged lateral ventricles in both hemispheres (Fig. 3A&B). The DAPI stained sections shown are at what we defined as brain level 5 using the Electronic Prenatal Mouse Brain Atlas (EPMBA) (Gestational day 16 coronal atlas as reference at epmba) [24]. Please refer to Table 1 for the approximate location of the atlas layers (sections) for each of the seven different levels we defined. To systematically quantify changes in the length and total region areas for major brain regions we sectioned brains from level 1 to 7 sections in EPMBA as detailed in Table 1. Length and brain areas were measured using Image-Pro Plus 3D Suite software revealed highly significant reduction in the cortical plate (CP) length at level 2 (31%, p<0.01), level 3 (47%, p<0.01), level 4 (38%, p<0.01) and level 5 (46%, p<0.01) in Ran−/− pups compared to littermate WT controls **(** Fig. 4A **)**. More striking differences were observed in the increased volumes of lateral ventricles (LV). During this period of mouse brain development, the lateral ventricles are hardly formed in the WT controls, but in the Ran−/− pups, already a huge ventricle can be observed. Thus, at level 2 (4694%, p<0.01), level 3 (1040%, p<0.001), level 4 (1968%, p<0.001) and level 5 (1731%, p<0.01) the lateral ventricles were significantly increased compared to littermate WT controls (Fig. 4B). Total cortical length was also significantly reduced at level 2 ((29%, p<0.01), level 3 (46%, p<0.001), level 4 (36%, p<0.01) and level 5 (44%, p<0.01) **(** Fig. 4C **)**. The marginal zone (MZ) showed significant difference only at level 1 (39%, p<0.001) and level 3 (34%, p<0.05) **(** Fig. 4D **)**. However, the length of the intermediate zone (IZ) was not altered at any of the levels examined (Fig. 4E). The total volume of the hippocampus (HI) was significantly reduced only at level 4 (49%, p<0.05) **(** Fig. 4F **)**.

**Table 1:** Levels 1–7 defined based on specific coronal layers (sections) in the Electronic Prenatal Mouse Brain Atlas (EPMBA).

Levels	Corresponding layer in the atlas	Approx distance from the origin in µm	Major anatomical hallmarks
1	53	530	Olfactory bulb, appearance of cortex
2	74	740	Lateral ventricle, appearance of caudate
3	127	1270	Lateral ventricle, caudate, appearance of choroid plexus
4	151	1510	Appearance of hippocampus, appearance of third ventricle
5	174	1750	Medium sized hippocampus
7	200	2000	Left and right neocortex derivatives separate
7	226	2260	Appearance of aqueduct, left and right neocortex derivatives decreases to half their size to the previous level

### Reduced Cortical Plate Length Reflects an Early Neonatal Requirement for RanBP9

**Figure 5:**
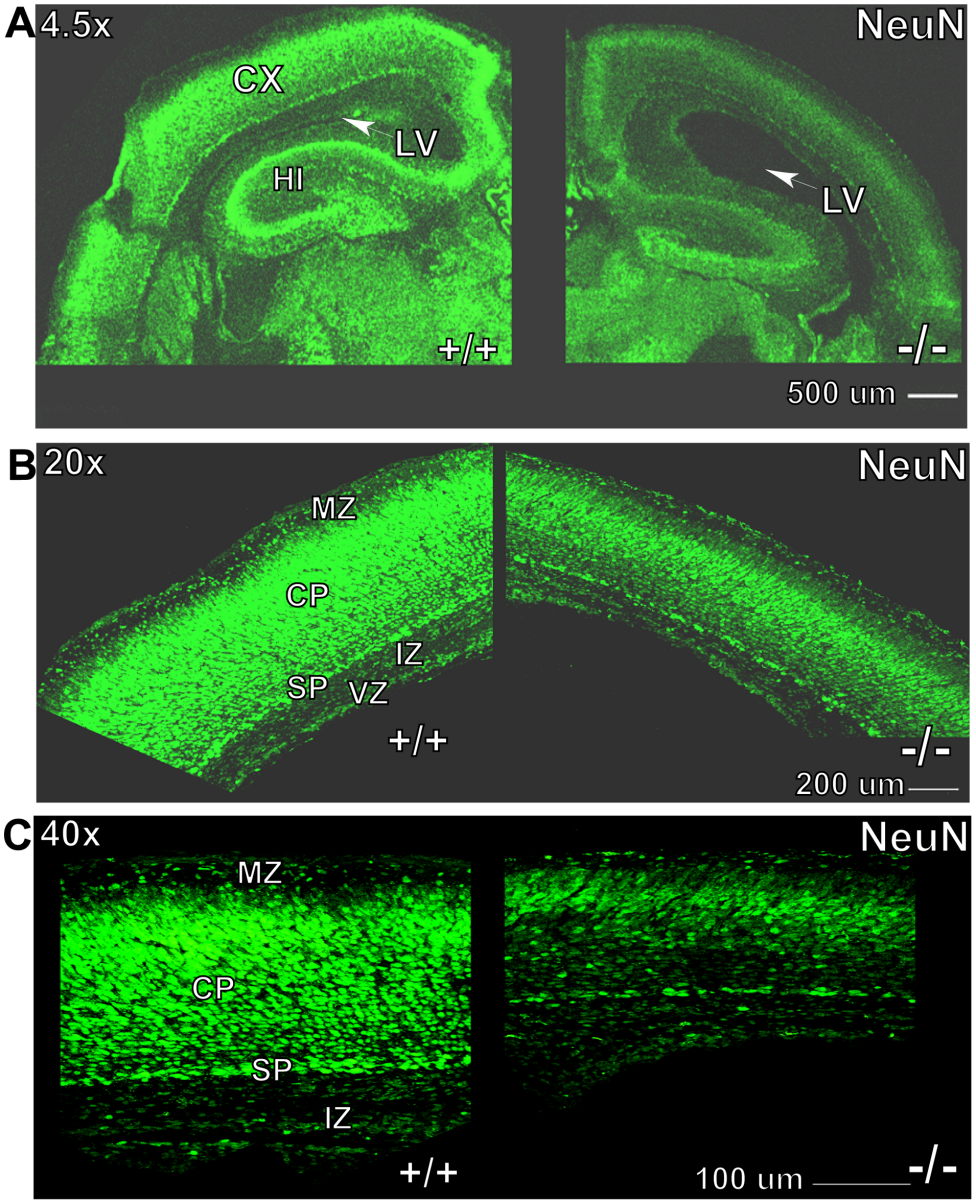
Confocal images of neuronal nuclear antigen (NeuN)-stained brain sections of WT and Ran−/− P1 mice at 4.5× (A), 20× (B) and 40× (C) as indicated. Note robustly increased lateral ventricular volume in Ran−/− brains at 4.5× (A), highly decreased thickness of cortical plate at 20× (B) and significantly reduced post-mitotic neurons at 40× (C) in Ran−/− brains compared to littermate WT (+/+) control brains. Scale bars are indicated for each magnification. CX, cortex, LV, lateral ventricle, HI, hippocampus, CP, cortical plate, SP, subplate, MZ, marginal zone, IZ, intermediate zone and VZ, ventricular zone.

**Figure 6:**
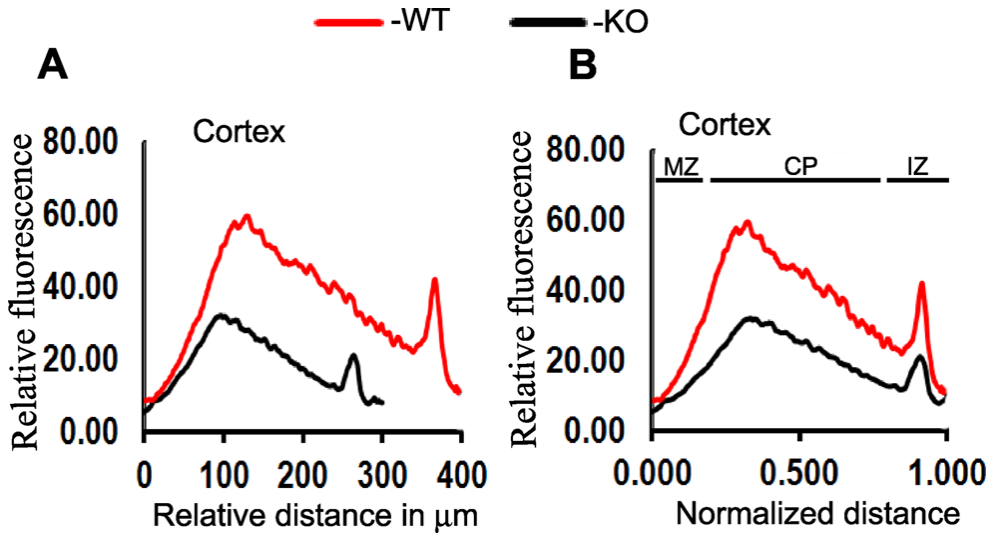
Relative fluorescence intensity of brain sections stained with NeuN and analyzed by Image-Pro Plus 3D Suite software in WT and Ran−/−P1 brains. A line profile analysis was performed for each area of interest (AOI) which generated a plot of the average intensity values. (A) Relative fluorescence intensity along the length of the cortex. Note the length differences in WT (400 µm) versus Ran−/− (300 µm) brains. (B) Fluorescence intensity normalized to the length of the cortex. The decreased fluorescence intensity is seen mostly in the cortical plate (CP) area and less so in the intermediate zone (IZ) and marginal zone (MZ).

RanBP9 protein can be detected both by immunoblots (Fig. 1C) and immunohistochemistry as early as postnatal day 0. Immustaining with RanBP9 specific antibody showed clear expression of RanBP9 protein in the MZ, CP, IZ, VZ as well as cells in other major brain regions (Fig. 3C) suggesting an essential role for RanBP9 in neonatal brain development. In contrast, Ran−/− brains did not reveal any immunoreactivity for RanBP9 antibody (Fig. 3D). Therefore to determine whether changes in brain length and area were due to fewer or smaller neurons, we next stained brain sections with an antibody against neuronal nuclear antigen (NeuN), a marker of mature post migratory neurons. High resolution pictures taken on a confocal microscope confirmed enlarged lateral ventricles and strikingly reduced CP length in Ran−/− pups compared to littermate WT pups (Fig. 5A). Superimposed confocal images also revealed that the numbers of neurons positive for NeuN were grossly reduced in Ran−/− P1 pups compared to WT pups **(** Fig. 5A, B&C **)**. It is also apparent that the fluorescence intensity in cells positive for NeuN in Ran−/− pups was also reduced consistently thought the brain implying faint expression of NeuN protein in these mice in contrast to robust expression in the WT brains. Quantification of relative fluorescence by Image-Pro Plus 3D Suite software throughout the length of cortex and hippocampus also revealed remarkable differences. Because of differences in the sizes of the cortex in Ran−/− pups versus WT, we normalized the fluorescence intensity to the distance and calculated the relative fluorescence in Ran−/− versus WT pups. Calculations revealed about one-fold reduction in fluorescence intensity in Ran−/− pups **(** Fig. 6A **)**. Interestingly, the decreased fluorescence intensity is accounted mainly by decreased cell density in the CP area as revealed by data for the normalized distance **(** Fig. 6B **)**.

### Reduced PCNA-positive Cells in the Dentate Gyrus of RanBP9−/− Brains

**Figure 7:**
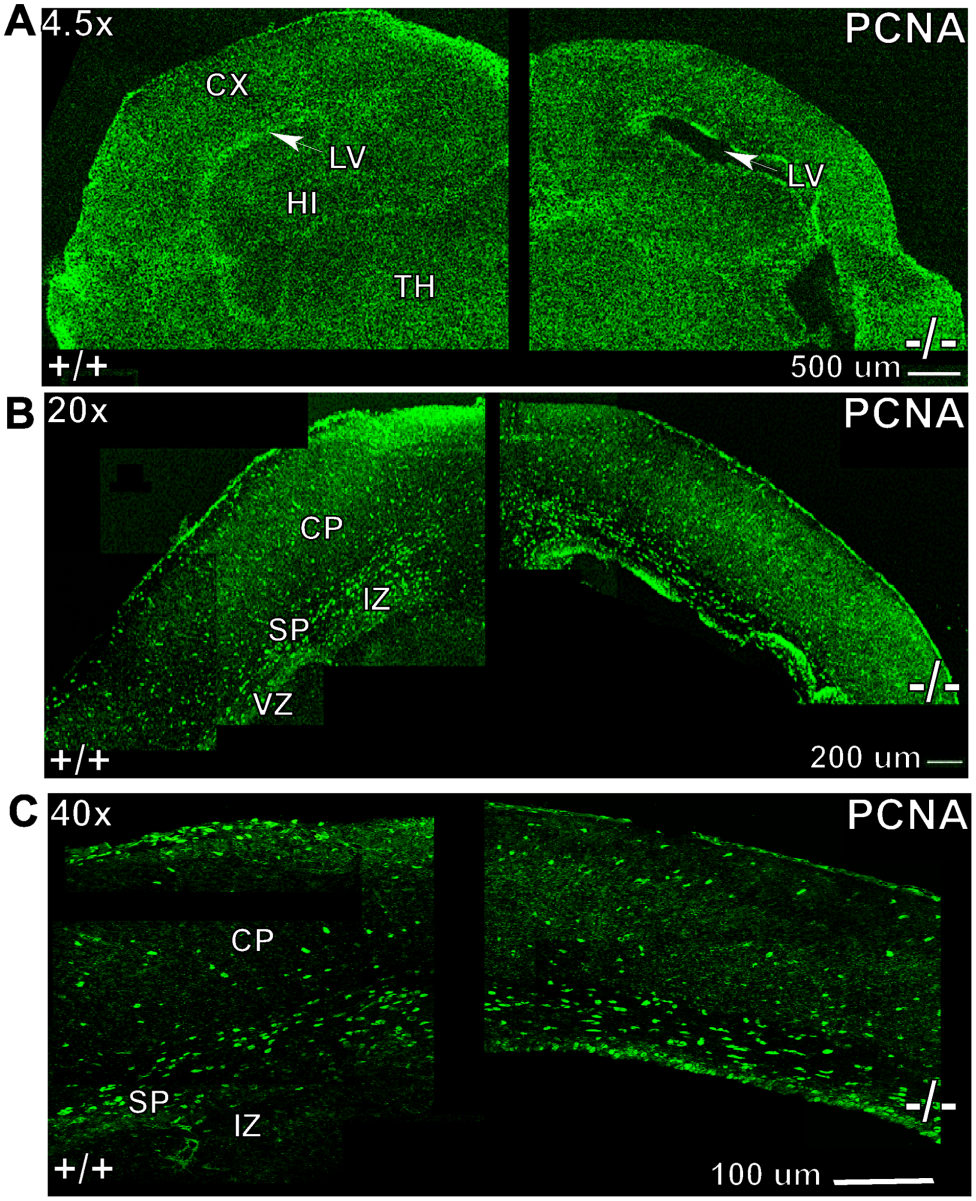
Confocal images of proliferating cell nuclear antigen (PCNA)-stained brain sections of WT and Ran−/− P1 mice at 40× as indicated. Note the number of cells positive for PCNA, however they were not different in Ran−/− brains compared to littermate WT (+/+) brains. Scale bars are indicated for magnification. CP, cortical plate, SP, subplate, IZ, intermediate zone.

To test whether reduced neuron density is due to reduced proliferation, we next stained brain sections of P1 mice with proliferating cell nuclear antigen (PCNA) to quantify the number of cells in the cell cycle. Cortical images of at least 3 sections per animal were imaged by confocal microscope and then the images were stitched together. Confocal images analyzed at 40× did not show any apparent difference in the staining pattern for PCNA between Ran−/− and WT brains (Fig. 7).The number of cells positive for PCNA were counted within an area of interest comprising the motor cortex of about 200 µm diameter between the subplate and the marginal zone, the Ran−/− brains showed no significant difference from WT littermate controls (Table 2). Thus RanBP9 does not appear to affect brain growth by inhibiting the cell cycle.

**Table 2:** Quantification of PCNA positive cells in brain sections of P1 mice corresponding to level 5 from wild-type and Ran−/− brains in a defined area at 40× magnification.

WT					
Mouse #	High intensity cells	Low intensity cells	Total cells	Area µm2	Normalized(60,000 µm2)
1	20	28	48	84179	34
2	19	27	46	79762	35
3	17	25	42	75373	33
Av.	19	27	45	79771	34
**Ran−/−**					
1	15	24	39	64367	36
2	17	19	36	62769	34
3	14	23	37	60939	36
Av.	15	22	37	62692	36

**Figure 8:**
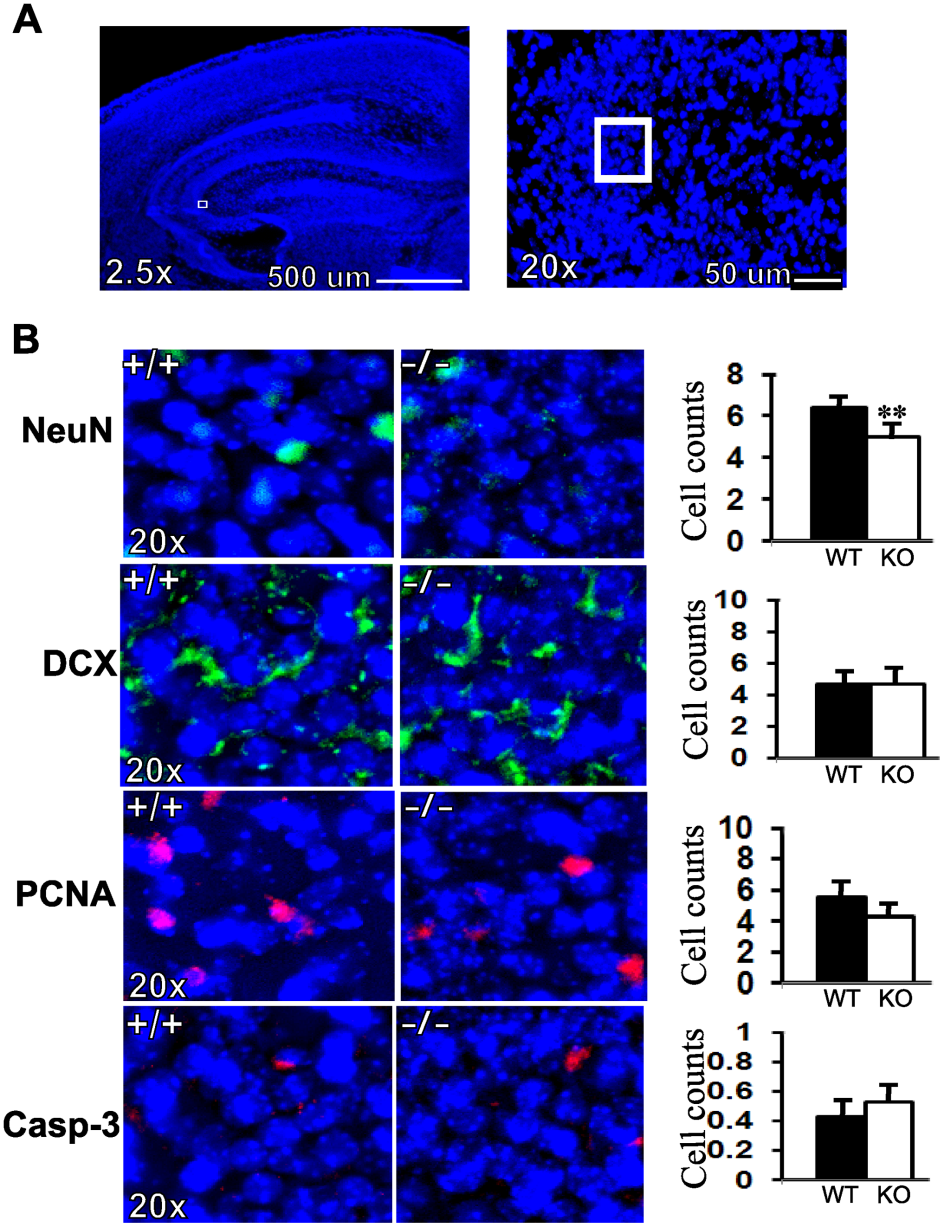
Cell numbers positively stained for NeuN, DCX, PCNA and caspase-3 in the subgranular region of the dentate gyrus in WT and RanBP9−/− (KO) mice. (A), DAPI-stained brain sections to show the highlighted subgranular zone within the dentate gyrus region of the hippocampus used for cell counts shown in B. (B), Representative brain sections stained with anti-NeuN, anti-DCX, anti-PCNA, anti-capsase-3 and counter stained with DAPI. Cell counting revealed significantly decreased numbers of NeuN positive cells in RanBP9−/− (KO) brains (22%) compared to WT controls. However DCX, PCNA and caspase positive cell numbers were not significantly altered. In each group, n = 3, data presented as mean± SEM. **, p<0.01 by Student’s t-test.

**Figure 9:**
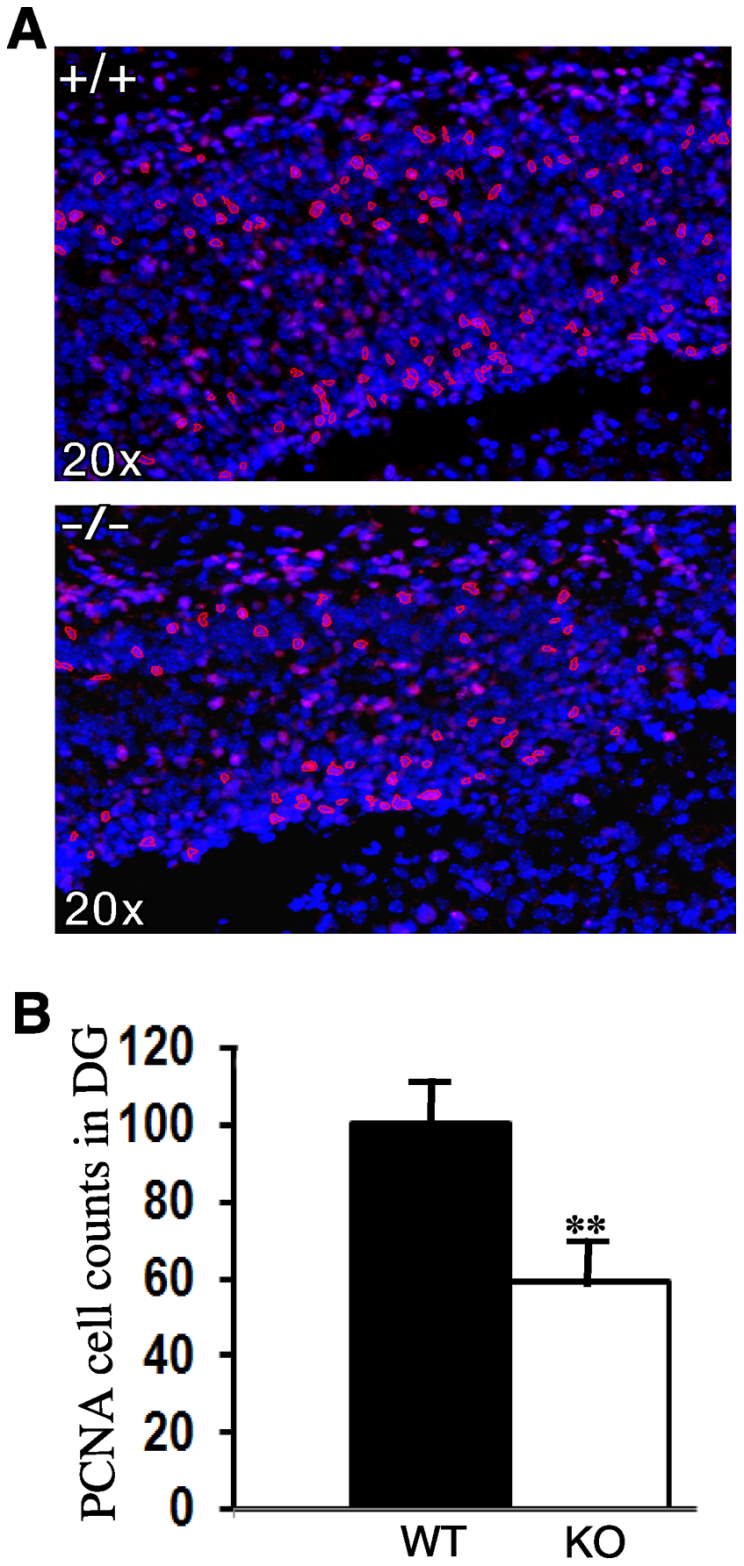
Cells positively stained for PCNA in the whole dentate gyrus region of the hippocampus in WT and RanBP9−/− (KO) mice. (A). Representative brain section to show whole of the dentate gyrus stained with PCNA (red) and DAPI (blue) in the WT (+/+) mice and the RanBP9 KO (−/−) mice. (B), Quantitation of PNCA-positive cells showed an average of 100 cells in the WT mice compared to only 59 in the RanBP9−/− mice which was statistically significant. In each group, n = 3, data presented as mean± SEM. **, p<0.01 by Student’s t-test.

To obtain more accurate cell numbers, we quantified the number of cells positive for NeuN, PCNA, doublecortin (DCX) and caspase-3 in a defined area measuring 50 µm × 50 µm in the subgranular zone of dentate gyrus within the hippocampus (Fig. 8A). We used automated cell counting using Image-Pro Plus software as well as manual counting. Since both methods gave identical numbers, we present the data for automated counting. Quantitation of NeuN-positive cells revealed a reduction of about 22% (p<0.01) in RanBP9−/− mice compared to WT controls (Fig. 8B). Although a decreasing trend was observed for PCNA-positive cells, there was no significant differences between RanBP9−/− and WT brains (Fig. 8B). Similarly, the DCX-positive and caspase-3-positive cells were identical in numbers in the RanBP9−/− and WT brains (Fig. 8B). Thus the cell numbers suggest that neuronal differentiation is defective more than proliferation in RanBP9 mice which may be responsible for the observed defects in suckling and subsequent lethal phonotype. Since quantitation in a selective and restricted area is liable to be biased and may not represent the whole brain region, we also quantified PCNA-positive cell numbers in the entire dentate gyrus region. Thus, when PCNA-positive cells were counted in the whole dentate gyrus, the WT brains showed an average of 100 cells compared to only 59 cells (p<0.01) in the RanBP9−/− brains (Fig. 9A&B). Therefore it is possible that overall proliferation may be significantly reduced in the Ran−/− brains compared to age-matched WT controls.

### Evidence for Reduced ERK1 Based Signaling in Ran−/− Pups

**Figure 10:**
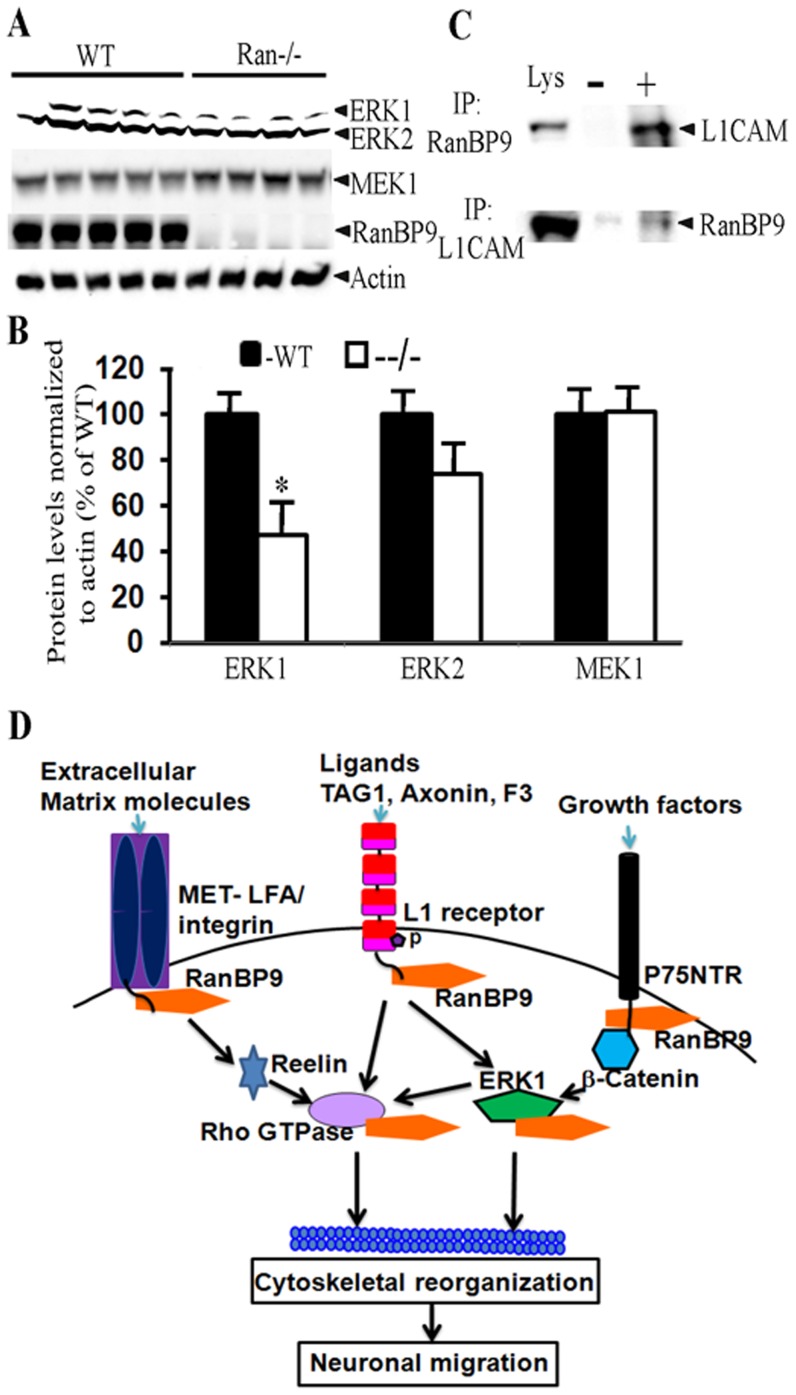
ERK1 protein levels are significantly reduced in Ran−/− brains from P1 mice. (A), Brain lysates from WT and Ran−/−mice were subjected to SDS-PAGE electrophoresis and ERK1/ERK2 & MEK1 proteins were detected using their specific polyclonal antibodies. Actin blot is shown as loading control and RanBP9 blot shows complete absence of RanBP9 protein in Ran−/− pups. (B) Quantitation of signal intensities by imageJ revealed significantly reduced protein levels for ERK1 (47%). n = 5 (WT) and 4 (Ran−/−),±SEM. *, p<0.05 by t test. (C), Endogenous L1CAM was pulled down by RanBP9 antibody (upper panel) and in a reciprocal coimmunopreciptation experiments, RanBP9 was pulled down by L1CAM antibodies (lower panel) suggesting that both L1CAM and RanBP9 interact with each other. (D), A model to explain mechanism of brain growth defects in Ran−/− brains. In response to cell adhesion signals, RanBP9 scaffolds L1CAM and ERK1 thereby activating downstream effectors such as Rho GTPases. Rho GTPases are known to physically interact and modulate cytoskeletal elements, which in turn alter neuronal differentiation/migration. RanBP9 is also known to bind MET-LFA integrin and p75NTR suggesting that alternative pathways to modulate differentiation/migration are also possible.

Numerous pathways have been implicated in the regulation of mouse brain growth and development [25]–[27]. Careful analysis of Ran−/− phenotype revealed marked similarities with ERK1/ERK2 double knockout mice [28]. Both Ran−/− pups and ERK1/ERK2 double knockout pups generally die within 24 h, both have no milk in the stomach, show cortical abnormalities and the pups are born reddish and appeared normal just after parturition ruling out abnormalities in either the cardiovascular or respiratory systems. Therefore we suspected that ERK1/ERK2 signaling may be impaired in Ran−/− brains. In fact, quantification of ERK1, ERK2 and MEK1 protein levels normalized to actin levels in Ran−/− brains confirmed significant reduction of ERK1 (53%, p<0.05) but not MEK1 levels **(** Fig. 10A&B **)**. Although ERK2 levels were reduced by 26%, it was not statistically significant.

It is also important to note that endogenous RanBP9 and L1CAM proteins physically interact with each other as demonstrated by reciprocal coimmunoprecipitation experiments (Fig. 10C). Like ERK1/2 double knockout mice, L1CAM knockout mice also share many phenotypic features. Thus, the identical phenotype of Ran−/− pups might result from altered L1CAM/ERK1 signaling in the brain. Our model to explain the mechanism of RanBP9-induced brain growth defects considers L1CAM and ERK1 to play major roles. Under normal conditions, in response to extracellular signals L1CAM is phosphorylated which activates downstream MEK1 and ERK1 signaling. Since RanBP9 binds both L1CAM and ERK1, it is possible that RanBP9 forms a scaffold bringing together functionally relevant proteins to activate the downstream effectors such as Rho GTPases which are known to modulate cytoskeletal reorganization, a required step for neuronal differentiation and migration (Fig. 10D). In the absence of RanBP9, both neuronal differentiation and migration may be severely affected leading to defects in cortical plate development, eventually leading to neonatal death.

## Discussion

Here we report behavioral phenotype as well as changes in brain anatomy associated with targeted deletion of the RanBP9 gene. The most obvious behavioral phenotype observed was lack of milk in the stomach suggestive of defects in suckling which leads to neonatal lethality of most pups within 24 h. Few pups that survived very rarely to about three weeks showed severe defects in hind limb coordination and posture during locomotion. Ran−/− brains showed an overall decreased number of neurons throughout the brain but the decrease was particularly remarkable within the cortical layers and also in the hippocampus.

Analysis of embryos on E18 revealed expected Mendelian ratios of Ran+/−, Ran−/− and WT genotypes suggesting that RanBP9 is not required for embryonic development. The fact that Ran−/− pups acquired pink color and were alive for several hours following parturition indicates that the neonatal lethality does not result from either respiratory or cardiovascular defects which normally results in death immediately after the birth. A detailed anatomical examination of pups on postnatal day 1 did not reveal gross differences in any of the visceral organs between Ran−/− and WT pups. Thus defects in non-neuronal tissues are unlikely responsible for the lethal phenotype. The most probable cause for neonatal lethality is lack of nourishment and loss of bodily homeostasis resulting from absence of fluid intake through the milk. Complex interactions between sensory and motor pathways are responsible for suckling behavior which involves nipple attachment and sucking movements. Since there is overall thinning of the cortex in Ran−/− brains both somatosensory and motor systems are expected to be defective. Neurogenesis in the developing mouse brain is temporally and spatially controlled, starting from E11.5 ventrolaterally and E12.5 dorsolaterally until E17.5 [29], [30]. Among the three distinct layers of cells apparent in the developing brain, i.e. CP, IZ and MZ, the most apparent thinning in Ran−/− pups occurs in the CP. As the ventricular volume and the cell density in the surrounding layers are inversely proportional, robustly increased lateral ventricle volume is due to thinning of cortical layers. In the mice, corticogenesis is a highly complex and coordinated process involving cell division, migration, layering, differentiation and finally apoptosis [29], [31]–[33]. We did not find any difference in thickness or the number of cells in the VZ, where most cells are formed in the Ran−/− brains compared to WT. Also, the neurons destined to the cortex are formed in the VZ prior to E17 [33], [34]. Lack of feeding by pups also suggests that olfactory bulb dysfunction with reduced number of olfactory neurons also might contribute to lethal phenotype. In a recent study, using RNAi based RanBP9 knockdown Chang et al [21] demonstrated that decreased RanBP9 expression rather increased the number of precursor cells in mitosis, by decreasing cells entering cytokinesis. Our PCNA data also suggested that the number of cells dividing in Ran−/− pups did not differ from WT controls. Thus defects in cell cycle and proliferation in the VZ are less likely the contributors to the thinning CP. Though our PCNA data suggested no changes in the number of cells dividing in Ran−/− pups, when quantified in the entire dentate gyrus region of the hippocampus, there was significant reduction in the PCNA-positive cells in the Ran−/− brains relative to the WT controls. This might suggest that defects in proliferation may be at least partially responsible for the observed phenotype. Additionally, significant reduction of NeuN-positive cells but not DCX-positive cells in the subgranular region of dentate gyrus in the Ran−/− brains suggests that defects in early-stage but not late-stage neuronal differentiation might also contribute to the phenotype.

On the other hand, migration of post-mitotic neurons into the CP continues until post-natal day (P) 3 [33], [35]. Since Ran−/− pups die within 24 h after birth when migration of post-mitotic neurons are at its peak, defects in migration might be a major contributing factor for the thinning of CP and therefore the neonatal lethality. The support for this notion comes from the observation that overexpression of RanBP9 in human renal carcinoma cell line; A704 increased the cell migration [9]. Therefore complete absence of RanBP9 in the developing brain is expected to severely limit migration of post-mitotic neurons leading to impaired corticogenesis and neonatal lethality. Although most Ran−/− pups died immediately after birth, some mice survived to term and even reached adulthood in another recent study which also produced Ran−/− mice [22]. In our experience, very few Ran−/− pups survived for a maximum of three weeks only. The reason for this discrepancy is not known, though like their strategy, we used the same gene-trap clone targeting the first intron of RanBP9 gene to produce Ran−/− pups. Similar to their finding that at embryonic day 17.5, Ran−/− mice were present at their expected frequency, we also found expected frequency of Ran−/− pups at embryonic day 18. This suggests that RanBP9 is not required for the embryonic development in mice. Our results differ from theirs mainly in the postnatal growth period. While they reported more than 60% lethality, we observed almost all of them die within postnatal day 1. Like ours, their study also found normal bodily organs in both size and histology including the testes on postnatal day 1. While they reported decrease in size of testes starting from postnatal day 8, pups in our hand did not survive beyond postnatal day 1 to make such comparisons. In our cell cultures also it was impossible to generate cells with a stable knockdown of RanBP9. Other laboratories have also reported failure to generate long-term knock down of RanBP9 in cells [13].

The striking similarity in phenotypes between Ran−/− and ERK1/2 double knockout mice as well as L1CAM knockout mice led us to suspect that ERK1/2 and L1CAM pathways may be involved in the observed phenotype. Our immunoblot confirmation that ERK1 protein levels are significantly reduced in Ran−/− brains clearly indicates that RanBP9 may be an upstream molecular link to the ERK pathway. Indeed, RanBP9 has been suggested to act as a scaffold for several receptors that network with the ERK1/2 pathway [9], [16], [36]. The ERK pathway in turn is activated by the neural cell adhesion protein L1CAM which regulates neurite outgrowth and most importantly migration of neuronal precursors [37]. Significantly, RanBP9 also bound endogenous L1CAM receptor by reciprocal coimmunoprecipitations and has been shown to regulate neurite out growth [16]. Thus RanBP9 acts as a scaffold bringing together functionally related proteins such as ERK1 and L1CAM to regulate physiological function such as neurite outgrowth and migration. In fact, we recently demonstrated that when RanBP9 is overexpressed in primary neuronal cultures, neurite outgrowth and branching were significantly reduced probably through integrin-dependent focal adhesion signaling [38]. Similarly we previously demonstrated that by scaffolding APP/BACE1/LRP complexes together, RanBP9 regulates endocytosis of APP in varieties of cell lines [39]. Interestingly, like Ran−/− brains L1CAM knockout mice also showed enlarged ventricles and reduced corticospinal tracts in addition to other abnormalities in cerebellum and corpus callosum [40]. Thus RanBP9, ERK1 and L1CAM bind with each other and regulate important cell processes in the same signal transduction pathway which may be responsible for similar phenotypes observed when each of these genes are functionally deleted. These data when taken together suggests that RanBP9 acts as an important scaffolding protein bringing together ERK1 and L1CAM proteins thereby regulating neurite outgrowth and/or neuronal migration which are critical physiological processes for proper brain growth and survival. Loss of RanBP9 reduces signaling through L1CAM receptors and ERK1 thereby adversely affecting migration and differentiation of post-mitotic neurons resulting in subnormal brain development which in turn leads to neonatal lethality.

In summary, we provided compelling evidence for the first time that RanBP9 plays a critical role in neonatal mouse brain development especially in the growth of the cortical plate and hippocampus. This subnormal brain growth appears to be responsible for the behavioral defects in suckling which in turn may lead to the observed neonatal death.

## Materials and Methods

### Ethics Statement

All animal procedures were carried out in strict accordance with the National Institute of Health’s ‘Guide for the Care and Use of Animals’ and approved by the Torrey Pines Institute’s Animal Care and Use Committee (IACUC).

### Generation of RanBP9 Null Mice by Gene-trap Strategy

Gene-trapping is a method to introduce mutations into embryonic stem cells (ES) by inserting a gene-trap vector construct through electroporation. Since gene-trap vectors produce varieties of insertional mutations, out of three insertional mutant clones available for RanBP9 from MMRRC, we obtained a mutant ES clone called RHA056 (IST 10422C6BBF1) because this clone had the insertion of the cassette within the first intron after the first exon at the farthest 5′ end of the gene. The ES cells were then expanded, tested for mycoplasma and other pathogens and karyotyped for the presence of normal set of chromosomes. The quality of ES cells was also verified for undifferentiation and correct shape of cells. Several aliquots of ES cells were stored in liquid nitrogen as a backup. Blastocyst injections were carried out at the Transgenic and Gene Targeting Core Facility, University of California, San Diego. Blastocysts were retrieved from a superovulated, mated C57Bl/6 mouse strain obtained from a commercial supplier. About 8–10 ES cells were injected into each of 30–40 blastocysts. Injected embryos were implanted into pseudopregnant mice and pups were borne after the 17 day gestation period.

Out of a total of 82 pups born from 10 mice, we identified 6 mice based on coat color (white patches), 3 males were 80–90% chimeric and another 3 females were 20–30% chimeric. All the 6 chimeric mice were weaned at 3 weeks and bred with C57Bl/6 mice at 6–8 weeks to achieve germline transmission. Fortunately, all the chimeras were fertile and produced many progenies over 2–3 mating cycles. Although, the mice with 20–30% chimera were less likely to go germline, we have obtained agouti mice from both the 20–30% chimeras as well as 80–90% chimeras. We obtained a total of 12 agouti mice. The mice with germline transmission were back crossed for several generations with C57Bl/6 mice, confirmed stable germ line transmission and the pure heterozygous mice (RanBP9+/−) were generated. The transmission of the RanBP9 null allele followed the Mendelian ratio suggesting that the integration site may be only one. Eventually, RanBP9−/− mice were generated by mating heterozygous mice.

As the sentinels in the mouse colony were tested positive for Murine Norovirus (MNV) and Helicobacter, we re-derived the RanBP9−/− mice at the Charles River Laboratories International Inc. (Wilmington, MA, USA) before using them for data acquisition presented in this manuscript.

### Confirmation of the Identity of the Trapped Gene

The gene-trap vector has a splicing acceptor (SA), gene-trap cassette which includes a selectable marker, a reporter and a poly A signal for the termination of the transcription. Since the insertional mutation creates a fusion transcript of sequences from exons upstream of the insertion and the βgeo marker, 5′ RACE followed by direct sequencing can be used to determine the identity of the trapped gene. A cDNA library was made from the ES clone, RHA056 using commercially available reverse transcriptase enzyme. A PCR reaction was performed using a forward primer in the Bay Genomics sequence tag and the reverse primer with the following sequence 5′-GAC AGT ATC GGC CTC AGG AAG ATC G-3′ within the β-galactosidase reporter. The PCR yielded a single band as expected and the specificity was confirmed by digestion with PvuII which released a 147 bp fragment from the β-galactosidase gene. The insertion of RHA056 within the first intron of RanBP9 gene was further confirmed by sequencing.

### X-gal Staining

To detect β-galactosidase activity, X-gal (5-bromo-4-chloro-3-indolyl-β-D-galactopyranoside) staining was carried out on coronal sections (15 µm). Sections were fixed in 0.1 M phosphate buffer supplemented with 5 mM EGTA, pH 7.3, 2 mM MgCl2 and 0.2% glutaraldehyde for 15 min at room temperature. The sections were washed twice, 5 minutes each with X-gal wash buffer and 0.1 M phosphate buffer (pH 7.3) supplemented with 2 mM MgCl2 and stained overnight in X-gal staining buffer containing 1 mg/ml X-gal.

### Tissue Extraction and Immunoblotting

We extracted whole brain tissue from Ran−/− and Ran+/− neonatal day 1 pups and littermate wild-type controls for immunoblot detection of RanBP9 (anti-RanBP9, monoclonal) protein, Ran-β-geo mutant protein using anti-β-galactosidase antibody (cat # ab616, Abcam, Cambridge, MA, USA) as well as ERK1/2 (cat # 4370, Cell Signaling, Danvers, MA, USA), MEK1 (cat # 9124, Cell Signaling, Danvers, MA, USA) and L1CAM (cat # NBP1-51294, Novus Biologicals) proteins using indicated antibodies. In brief, we anesthetized the mice with isoflurane, decapitated immediately and rapidly removed the brain tissue in to 1% NP40 buffer (50 mM Tris-HCl, pH 8.0, 150 mM NaCl, 0.02% sodium azide, 400 nM microcystine-LR, 0.5 mM sodium vanadate and 1% sodium Nonidet P-40) containing complete protease inhibitor mix (Sigma, St. Louis, USA). Tissue was homogenized using Power Gen 125 (Fisher Scientific, Pittsburgh, USA) and centrifuged at 100,000 g for 1 hr. Protein concentrations from each sample were measured by BCA method (Pierce Biotechnology Inc., Rockford, USA). Equal amounts of proteins were loaded into each well and subjected to SDS-PAGE electrophoresis. The proteins were then transferred onto PVDF membranes, blocked with 5% milk and incubated overnight with primary antibodies followed by one hour incubation with HRP-conjugated secondary antibodies such as monoclonal mouse anti-Goat IgG light chain (cat # 205-032-176, Jackson ImmunoResearch, West Grove, PA, USA) or monoclonal mouse anti-Rabbit IgG light chain (cat # 211-032-171, Jackson ImmunoResearch, West Grove, PA, USA). The protein signals were detected using Super Signal West Pico Chemiluminescent substrate (Pierce, USA).

### Immunohistochemistry

Neonatal Ran−/−, Ran+/− and littermate WT P1 pups were deeply anesthetized using isoflurane. The brains were carefully removed from the skull as quickly as possible avoiding any damage, immediately immersed in a 4% paraformaldehyde solution (PFA) (Fisher Scientific, Pittsburgh, PA, USA) in PBS with gentle rocking at 4°C for 36–48 hours to fix the brain tissue. After removing PFA, brains were incubated in 1× PBS with constant rocking at 4°C overnight, followed by incubation with sucrose gradients (10%, 20%, and 30%) at 4°C. Dehydrated brains were then embedded in Optimal Cutting Temperature compound (OCT, Tissue-Tek), frozen on dry ice in cold 2-methyl butane (Sigma, St. Louis, USA), and stored at −80°C. Frozen brains were cut on the cryostat (Leica, model CM1850), 14 µm sections were placed on subbed slides and frozen at −30°C overnight. Antigen retrieval was carried out by immersing thawed slides in 10 mM citric acid, pH 6.0 (Acros Organics) for 10 min at 80–90°C. Sections were washed with 1× PBS for 5 min 2 times, and incubated in blocking solution for 1 h at room temperature. The following primary antibodies were diluted in blocking solution (1∶100) and added to the sections for 24–36 h at 4°C: NeuN (cat # IHCR 1001-6, Millipore, Billerica, MA, USA), proliferating cell nuclear antigen (PCNA) (cat # 3350R-100, Biovision, Milpitas, CA, USA), and RanBP9 monoclonal antibody (21–23). After 3–5 min washes in 1× PBS, the sections were incubated at room temperature for 2 h in the dark with the following secondary antibodies in blocking solution (1∶200): Alexa Fluor 488 goat anti-mouse IgG (Life Technologies, Grand Island, NY, USA), Alexa Fluor 488 goat anti-rabbit IgG (Life Technologies, Grand Island, NY, USA). Finally, slides were washed 4 times with 1× PBS for 5 min each, covered with mounting medium for fluorescence with DAPI (Vector Laboratories, Burlingame, CA, USA) and sealed with clear nail polish. Sections were visualized under an Axio Examiner D1 microscope (Zeiss, Standort Göttingen – Vertrieb, Deutschland) and pictures were taken using AxioCam HRc and AxioCam MRm cameras controlled by Axiovision software.

### Cell Counts

Brains of 3 wild type and 3 RanBP9−/− mice were dissected on postnatal day 1 (P1). Coronal sections (level 5) were immunohistochemically stained for DCX (cat # ab18723, Abcam, Cambridge, MA, USA), NeuN (cat # MAB377, Millipore, Temecula, CA, USA), PCNA (cat# 3350R-100, BioVision, Milpitas, CA, USA), and Caspase 3 (cat # NB100-56708, Novus Biologicals, Littleton, CO, USA) and co-stained with DAPI. Alexa Fluor 488 (cat # A11008, Invitrogen, Carlsbad, USA) was used for NeuN and DCX, while Alexa Fluor 568 (cat # A-11011, Invitrogen, Carlsbad, USA) was used for PCNA and Caspase 3. Images were taken at 20×. An area of interest (AOI) of 50×50 µm was defined within the subgranular zone of the dentate gyrus (DG). The images at the AOI were projected, flattened, and their histograms were normalized using Image Pro Plus Suite software. A high intensity threshold was set which was followed by automated cell quantification so that only cells with high marker expression were quantified (only objects above a defined intensity in the AOI were considered). Images were merged with DAPI stained nuclei with the exception of DCX, which was quantified manually, since it was only observed in the cytoplasm, mostly in dendritic branches. Neurons were also manually traced.

### Confocal Microscopy and Imaging

For images stained with NeuN and PCNA, we also used a laser-scanning confocal microscope (Nikon 90i C1 SHS, Melles Griot laser system). Images were deconvoluted, filtered, and analyzed with advanced image analysis software, Image-Pro Plus 3D Suite (Media Cybernetics, Bethesda, MD, USA). Some pictures were montaged and fluorescence intensity was quantified using Image-Pro Plus software. To quantify fluorescence intensity, brain sections that were probed with only secondary antibodies lacking the primary antibody was used for background subtraction. A line profile analysis was performed for each area of interest which generated a plot of the average intensity values. The values of 3 plots for three sections for each brain of either WT or Ran−/− were averaged, and subsequently the values of the 3 average plots for all three mice were averaged.

### Statistics

Signal intensities of Western blots were quantified using ImageJ software followed by Student’s t test. The fluorescence intensities of brain sections stained with NeuN and PCNA were analyzed by analysis of variance (ANOVA) followed by post-hoc tests using Instat3 software (GraphPad Software, San Diego, CA, USA). For t tests, we used two-tailed p value assuming populations may have different standard errors. The data were considered significant only if the p<0.05. * indicates p<0.05, **, p<0.01 and ***, p<0.001.
